# Fractal analysis improves tumour size measurement on computed tomography in pancreatic ductal adenocarcinoma: comparison with gross pathology and multi-parametric MRI

**DOI:** 10.1007/s00330-022-08631-8

**Published:** 2022-02-24

**Authors:** Florian Michallek, Mohamed Amine Haouari, Ophélie Dana, Antoine Perrot, Stéphane Silvera, Axel Dallongeville, Marc Dewey, Marc Zins

**Affiliations:** 1 Department of Radiology, Charité – Universitätsmedizin Berlin, corporate member of Freie Universität Berlin, Humboldt-Universität zu Berlin, and Berlin Institute of Health, Charitéplatz 1, 10117 Berlin, Germany; 2 Groupe Hospitalier Paris Saint-Joseph, 185 rue Raymond Losserand, 75014 Paris, France

**Keywords:** Carcinoma, Pancreatic ductal, Four-dimensional computed tomography, Multi-parametric magnetic resonance imaging, Perfusion imaging, Fractals

## Abstract

**Objectives:**

Tumour size measurement is pivotal for staging and stratifying patients with pancreatic ductal adenocarcinoma (PDA). However, computed tomography (CT) frequently underestimates tumour size due to insufficient depiction of the tumour rim. CT-derived fractal dimension (FD) maps might help to visualise perfusion chaos, thus allowing more realistic size measurement.

**Methods:**

In 46 patients with histology-proven PDA, we compared tumour size measurements in routine multiphasic CT scans, CT-derived FD maps, multi-parametric magnetic resonance imaging (mpMRI), and, where available, gross pathology of resected specimens. Gross pathology was available as reference for diameter measurement in a discovery cohort of 10 patients. The remaining 36 patients constituted a separate validation cohort with mpMRI as reference for diameter and volume.

**Results:**

Median RECIST diameter of all included tumours was 40 mm (range: 18–82 mm). In the discovery cohort, we found significant (*p* = 0.03) underestimation of tumour diameter on CT compared with gross pathology (Δdiameter_3D_ = −5.7 mm), while realistic diameter measurements were obtained from FD maps (Δdiameter_3D_ = 0.6 mm) and mpMRI (Δdiameter_3D_ = −0.9 mm), with excellent correlation between the two (*R*^2^ = 0.88). In the validation cohort, CT also systematically underestimated tumour size in comparison to mpMRI (Δdiameter_3D_ = −10.6 mm, Δvolume = −10.2 mL), especially in larger tumours. In contrast, FD map measurements agreed excellently with mpMRI (Δdiameter_3D_ = +1.5 mm, Δvolume = −0.6 mL). Quantitative perfusion chaos was significantly (*p* = 0.001) higher in the tumour rim (FD_rim_ = 4.43) compared to the core (FD_core_ = 4.37) and remote pancreas (FD_pancreas_ = 4.28).

**Conclusions:**

In PDA, fractal analysis visualises perfusion chaos in the tumour rim and improves size measurement on CT in comparison to gross pathology and mpMRI, thus compensating for size underestimation from routine CT.

**Key Points:**

• *CT-based measurement of tumour size in pancreatic adenocarcinoma systematically underestimates both tumour diameter (Δdiameter = −10.6 mm) and volume (Δvolume = −10.2 mL), especially in larger tumours*.

• *Fractal analysis provides maps of the fractal dimension (FD), which enable a more reliable and size-independent measurement using gross pathology or multi-parametric MRI as reference standards*.

• *FD quantifies perfusion chaos—the underlying pathophysiological principle—and can separate the more chaotic tumour rim from the tumour core and adjacent non-tumourous pancreas tissue*.

## Introduction

Computed tomography (CT) plays an important role in staging pancreatic ductal adenocarcinoma (PDA) in clinical routine. The current edition (8^th^) of the American Joint Commission on Cancer (AJCC) staging system for pancreatic adenocarcinoma [1] differs from earlier versions in that it introduces a size-based definition for T-staging. This size-based definition has been validated and prognostic implications have been derived [2–5]. However, it is also known that tumour size, measured either as diameter or volume, is frequently underestimated on CT compared with multi-parametric magnetic resonance imaging (mpMRI) or pathological workup of the resected specimen [6–9]. This phenomenon has been associated with the presence of a vital tumour rim, which in some cases can be evident as a slightly hyperperfused halo in comparison to surrounding pancreatic tissue, but in many other cases is not depicted on CT imaging [10]. This tumour rim seems to differ from the hypoperfused tumour core in terms of the observable perfusion pattern [11].

Fractal analysis is a technique to quantitatively describe perfusion patterns and has been applied to radiological perfusion imaging [12–14]. The quantitative result of fractal analysis—the fractal dimension (FD)—represents the amount of chaos in the perfusion pattern and can be related to the structure of the underlying vascular tree. Tumour neoangiogenesis patterns in PDA include the formation of impaired and poorly perfused, chaotically arranged blood vessels alongside vasculogenic mimicry, which determines the perfusion pattern in PDA [15–17]. Previous studies have observed higher perfusion rates in the rim of PDA than in the tumour core and have related this finding to a greater microvascular density in the rim [18, 19]. A histopathological explanation suggests that activated pancreatic stellate cells might play a role in upregulating relative vascular density in the tumour rim [20]. Exploiting perfusion pathophysiology, fractal analysis might unveil the different perfusion pattern in the tumour rim, thereby enabling more realistic measurement of tumour size on CT. This could improve clinical management of patients with PDA in three situations: (1) follow-up of patients with chemotherapy and in particular evaluation of response in patients undergoing neoadjuvant treatment; (2) improvement of accuracy for assessment of vascular invasion in upfront surgery; and (3) improvement of precision for radiation therapy planning [6, 21].

In this study, we hypothesise that fractal analysis enables visualisation of the vital, proliferatively active tumour rim by quantifying the chaos of the local perfusion pattern. We investigate whether fractal analysis allows more reliable measurement of tumour volume and diameter in patients with PDA by comparing the results of CT-based fractal analysis with gross pathology and mpMRI [9].

## Patients and methods

### Patients

A retrospective study was performed after obtaining approval from the institutional review board (IRB number 00012157) and written informed consent was waived. Clinical routine data were analysed and patients with histologically proven PDA either from surgery or biopsy were included if CT and MRI datasets acquired within 1 week at the time of diagnosis were available.

### Imaging protocols

CT imaging was performed in a single-centre, single-scanner (64 rows, Optima CT660, GE Healthcare) setting. Scanning parameters were as follows: helical scan mode with tube voltage of 120 kV and tube current of 140 mAs, 0.8-s gantry rotation time, pitch factor of 1.375, and 64 × 0.675 mm collimation. Images were reconstructed on 512 × 512 pixel image matrices with resolution on the order of 0.7 mm × 0.7 mm × 1.25 mm and 1.25-mm increment using a soft tissue convolution kernel. CT was performed in a non-enhanced phase and after intravenous administration of a non-ionic iodine-based contrast agent (iodine concentration of 300 mg/mL, iopromide, dose of 1.5 mL/kg injected at a rate of 3.5 to 4 mL/s, followed by a 40 mL saline flush at 3 mL/s). The scanning delay for pancreatic parenchymal phase imaging was determined by using a bolus-tracking technique with automated scan-triggering software (SmartPrep; GE Healthcare). Acquisition of the pancreatic parenchymal and hepatic venous phases was started automatically at 17 and 50 s, respectively, after the trigger threshold (100 HU) was reached at the level of the supracoeliac abdominal aorta [22].

All MRI examinations were performed on a single scanner (3 T, Discovery MR 750, GE Healthcare) using body phased array coils. The patients were imaged after an overnight fast. The following pulse sequences were obtained: 2D T2-weighted fat-saturation (FS) fast-recovery fast spin echo (FRFSE); pre-contrast 3D T1-weighted gradient echo (GRE); 3D T1-weighted FS dynamic GRE sequences in the arterial phase (25 s), portal phase (70 s), and delayed phase (3 and 5 min) after gadolinium-based contrast agent injection; and diffusion-weighted imaging (DWI) sequences (three directions, b0 combined with different gradients from the following set 50, 200, 400, 600, and 800 s/mm^2^). The contrast agent was either gadoteric acid (Dotarem, Guerbet) at a dose of 0.2 mL/kg or gadobutrol (Gadovist, Bayer Schering Pharma) at a dose of 0.1 mL/kg, injected at a flow rate of 2 mL/s and followed by a saline flush (40 mL at 2 mL/s). Apparent diffusion coefficient (ADC) maps were generated using a post-processing console (Advantage Windows) with a monoexponential decay model.

### Software, pre-processing, segmentation, and fractal analysis

The first author developed the fractal analysis software in Python 3.6, which implements the pre-processing pipeline including image registration and image standardisation as well as fractal analysis and segmentation. The CT imaging data were processed as follows. First, image registration was performed with the SimpleElastix framework, version 4.9 (https://simpleelastix.github.io/, Kasper Marstal) employing a standard combination of 3D affine and 3D b-spline transformations. Second, images were standardised for voxel size, image noise, and signal intensity. All images were resampled to an isotropic voxel spacing of 0.7 mm as a common denominator. To standardise image noise, a noise-level-adapted denoising scheme was employed consisting of a 3D median filter with a radius set to 2 pixels and a 3D bilateral filter [23], whose distance parameter was set to 1 pixel and range parameter defined according to image noise determined from a region of interest (ROI) placed in the spinal erector muscles. For intensity standardisation, the signal was standardised using the portal-venous signal intensity at each time point as follows. We measured unenhanced portal-venous signal (*I*_0_) and related each voxel’s signal (*I*_voxel_) in the contrast-enhanced phases to the respective portal vein signal intensity (*I*_pv_) to obtain a normalised signal as follows: *I* = (*I*_voxel_ − *I*_0_) / (*I*_pv_ − *I*_0_) × 100. This was done to standardise image intensity by a physiological reference, thereby accounting for potential differences in circulation and mitigating the issue of potential variation in timing image acquisition. After pre-processing, four-dimensional, local A fractal analysis was performed to generate maps of the local FD. A previously published method [24] was used and extended to 4D to integrate information from the unenhanced, pancreatic parenchymal and portal-venous phases of perfusion. This method moulds two “blankets” to image texture, one upper blanket and one lower blanket. The blankets are iteratively raised or, respectively, lowered from the original texture to evaluate fractal properties as a function of loss of detail. Fractal analysis yields FD as a quantitative parameter of geometrical complexity, or chaos, and takes fractional values between 4 and 5 in the four-dimensional application. For local fractal analysis, we evaluated each voxel’s immediate vicinity, i.e., its direct neighbours. Local FD maps were calculated for visualisation and segmentation. In addition to local fractal analysis, we performed 4D global fractal analysis by evaluating larger, anatomically coherent regions, i.e., tumour core, tumour rim, and remote pancreas. Unlike its local variant, global fractal analysis does not yield a local FD map, rather it yields a single FD scalar value for the whole ROI. Global FD was interpreted as an explainable measure of the global chaos of the perfusion pattern. Mathematical details of global fractal analysis have been described previously [25].

### Diameter and volume measurements

To characterise the patient cohort, tumour diameter was conventionally measured on CT using the revised RECIST recommendations [26], which reflects pre-operative size measurement in clinical practice. For further analysis, the tumours were volumetrically segmented by the first author (experience in abdominal CT: 6 years) using a freehand drawing tool and subjected to a consensus reading (senior authors, experience in abdominal CT: > 20 years) on CT, FD maps, and MRI. On CT, the visible tumour portion (usually the hypoperfused tumour core) was segmented using the phase with the best visual conspicuity, incorporating information from both contrast-enhanced phases. We performed image fusion of FD maps and CT images to ensure correct anatomical positioning when using local FD maps for size measurement. Segmentation was performed using FD discrepancies between tumour and adjacent pancreatic tissue. In MRI, we used DWI at the highest *b*-value (i.e., 800 s/mm^2^ in our study) and T2-weighted images for volumetric tumour segmentation and T1-weighted fat-saturated contrast-enhanced images to visually assess conspicuity [9]. Voxel spacing on DWI ranged between 1 and 1.6 mm in-plane and 5 mm in z-direction, which yields a precision of at least 1.6 mm × 1.6 mm × 5 mm = 12.8 mm^3^ = 0.128 mL. From tumour segmentations, volume and maximum diameter were calculated [27]. The maximum diameter, which was subjected to statistical analysis, is given in terms of Feret’s diameter, which represents the maximum tumour extent independently of spatial alignment or axis orientation. As such, it is a robust and objective method of diameter quantification, suffering less from inter-observer variability than conventional diameter measurement according to RECIST recommendations [27–30]. The largest tumour diameter on gross pathology examination served as the reference standard, where available, and was analysed in an initial discovery cohort. The findings in the discovery cohort were independently validated in the remaining patients as a separate cohort with intermodal size estimation comparisons.

### Statistical analysis

In the discovery cohort with gross pathology reference, diameter measurements were tested for linear correlation of CT, mpMRI, and fractal analysis with pathology ground truth. Differences in size estimation between each method and the reference standard were evaluated using the pairwise Wilcoxon rank sum test for paired samples and Bonferroni correction for multiple testing. In the validation cohort with mpMRI as reference, CT and fractal analysis measurements of tumour volume and diameter were evaluated for linear correlation with mpMRI as reference. Furthermore, the *t*-test was used to assess measurement differences per method. Bland-Altman statistics were calculated to evaluate systematic size measurement differences for each method, and the *f*-test was performed to evaluate variances of the differences. In a subpopulation of 20 cases, we assessed inter-reader agreement (two readers with 6 and > 15 years of experience in abdominal imaging) in terms of relative size measurement discrepancy (in %) and Spearman’s *ρ*. To test whether fractal analysis is capable of identifying the tumour rim, local chaos per anatomical region (i.e., tumour core, tumour rim, and adjacent non-tumourous pancreas) was analysed using descriptive statistics as well as the Kruskal-Wallis test and pairwise group comparisons using the Mann-Whitney *U*-test with Bonferroni correction to test for perfusion chaos differences between those anatomical sites. A level of *p* ≤ 0.05 (after Bonferroni correction where appropriate) was considered statistically significant. Statistical analysis was performed with R (v3.4.1; R Foundation for Statistical Computing).

## Results

### Patient characteristics and outcome of image processing

A total of 46 patients were retrospectively included. The median diameter of all included tumours was 40 mm (range: 18–82 mm). Tumour characteristics and imaging findings are summarised in Table 1. Image processing and fractal analysis were successful in all patients and took about 20 min per patient including registration (5 min), image processing including local and global fractal dimension calculation (1 min), and manual multimodal segmentation with volumetry and diameter measurement (CT: 5 min, FD map: 3 min, mpMRI: 5–10 min).

**Table 1:** Study cohort characteristics

Characteristic	Absolute number	Relative fraction
Total number	46	
Median age (range)	69 (43–86)	
Female sex	21	46%
Tumour diameter on CT *		
≤ 2 cm	14	31%
> 2 cm and ≤ 4 cm	23	44%
> 4 cm	9	25%
Tumour location within pancreas		
Head	29	63%
Body	12	26%
Tail	5	11%
Duct dilation		
Pancreatic duct	30	65%
Bile duct	30	65%
Vascular involvement		
CHA	13	28%
CA	12	26%
Abutment (< 180°): SMA, 1st jejunal branch or aorta	8	17%
Encasement (≥ 180°): SMA, 1st jejunal branch or aorta	6	13%
PV, SMV, SV	29	63%
Regional lymph nodes **		
None involved	18	40%
1–3 involved	14	30%
> 3 involved	14	30%
Extrapancreatic growth	11	24%

*CHA*, common hepatic artery; *CA*, coeliac axis; *SMA*, superior mesenteric artery; *PV*, portal vein; *SMV*, superior mesenteric vein; *SV*, splenic vein

^*^Note that the diameters given in this table are the maximum diameters depicted in the orthogonal standard planes in CT according to revised RECIST recommendations. **based on criteria in [26]

### Discovery cohort: comparison to gross pathology

**Fig. 1:**
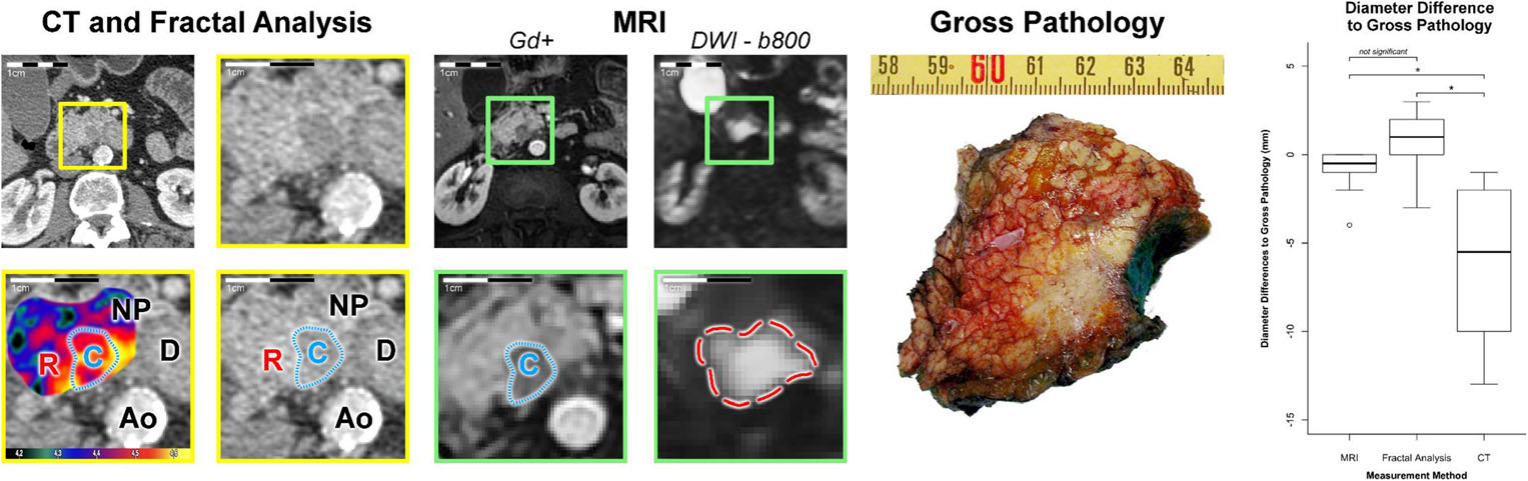
Representative example of pancreatic ductal adenocarcinoma in the pancreas head with gross pathology from the resected specimen as reference. CT with fractal dimension (FD) map of perfusion (left and middle columns with yellow frames, FD colour-code given in the bottom inset) and MRI as reference. A contrast-enhanced, fat-saturated T1 GRE image (acquired after administration of a gadolinium-based contrast agent) in the arterial phase (Gd+) is shown to illustrate tumour conspicuity alongside a diffusion-weighted image with *b*-value = 800 s/mm^2^ (DWI - b800), which was used for size estimation. The panels with yellow and green frames are magnifications of the tumour area in CT and MRI, respectively, and correspond to the marked areas in the images with original resolution. Gross pathology depicts a cross-section through the pancreas head and the tumour, which measured 25 mm in maximum diameter. Tumour diameter on CT was 15 mm, on FD map 26 mm, and on MRI 24 mm. The boxplot on the right summarises differences in diameter measurements between gross pathology as reference and each imaging estimation method. CT significantly underestimated tumour diameter with a mean difference of −5.5 mm (confidence interval: −9.5 to −1.5), whereas MRI and FD measurements did not significantly differ from gross pathology. *p = 0.03; C, tumour core; R, tumour rim; NP, non-tumourous pancreatic tissue; D, duodenum; Ao, aorta

Our initial discovery cohort consisted of 10 patients, in which diameter measurements on gross pathology were available as reference standard. Both mpMRI and FD size estimates correlated well with gross pathology measurements (mpMRI: *R*^2^ = 0.96; FD: *R*^2^ = 0.92) and did not show significant diameter differences (mpMRI: Δdiameter = 0.9 mm, *p* = 0.32; FD: Δdiameter = −0.6 mm, *p* = 1.0). Moreover, measurements on FD maps correlated excellently to mpMRI (*R*^2^ = 0.88) without significant differences between those methods (*p* = 0.63). In contrast, CT measurements systematically underestimated tumour diameter in comparison to gross pathology (Δdiameter = 5.7 mm, *p* = 0.03). A representative example with correlation to gross pathology is depicted in Figure 1 alongside a boxplot to summarise diameter differences measured on each modality to pathological reference. Detailed statistics for the discovery cohort are summarised in Table 2.

**Table 2:** Comparison of volume and maximum diameter measurements by method with gross pathology measurement as reference standard in the discovery cohort

Method	Bland-Altman statistics			Linear correlation with reference	
	Mean difference (lower and upper limit)	Slope of regression line (CI)	Intercept of regression line (CI)	Slope (CI)	*R*^2^
Diameter measurements (mm)					
mpMRI	0.9 (−1.62 to 3.42)	0.05 (−0.09 to 0.19)	−0.51 (−4.74 to 3.72)	0.94 (0.81 to 1.07)	0.97
FD	−0.6 (−4.43 to 3.23)	0.05 (−0.17 to 0.27)	−2.15 (−8.9 to 4.61)	0.92 (0.71 to 1.12)	0.92
CT	5.7 (−2.46 to 13.86)	0.07 (−0.42 to 0.56)	3.76 (−9.88 to 17.41)	0.78 (0.36 to 1.2)	0.65

Absolute volume or diameter measurements are given in ml or mm, where applicable. *mpMRI*, multi-parametric magnetic resonance imaging; *FD*, fractal dimension map; *CI*, 95% confidence interval; *R*^*2*^, coefficient of determination

### Validation cohort: tumour volume and diameter measurements

Since pathological reference was not available in our validation cohort (*n* = 36), we used mpMRI as surrogate due to its excellent correlation with gross pathology measurements as demonstrated in the initial discovery cohort. Tumour volume on FD maps correlated well with mpMRI volumetry (Δvolume = −0.6 mL, *p* = 0.86) and showed no size-dependent variation (*p* = 0.77). In contrast, tumour volume was consistently underestimated on CT in comparison to mpMRI (Δvolume = 10.2 mL, *p* < 0.001) with the amount of underestimation increasing with absolute tumour size (*p* = 0.01).

**Fig. 2:**
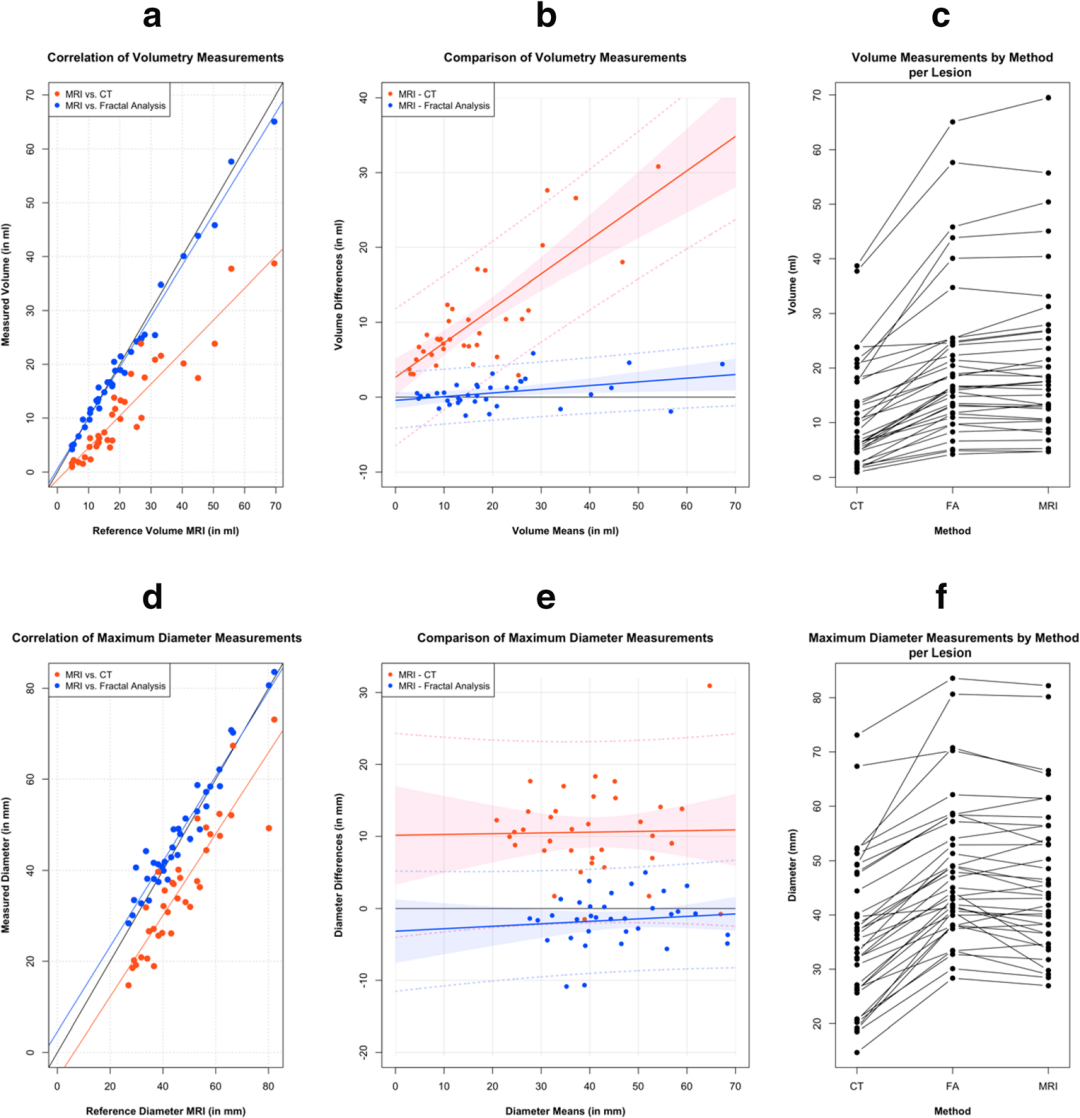
Correlation and agreement of volume (**a**–**c**) and maximum diameter (**d**–**f**) measurement methods with multi-parametric MRI (mpMRI) as reference. **a** and **d** show a plot of the linear correlation of CT and fractal dimension (FD) mapping with mpMRI (for quantitative evaluation see Table 2). Bland-Altman plots by method for volume and maximum diameter are depicted in **b** and **e**, respectively, with the difference being defined as MRI measurement (reference) minus candidate test measurement (i.e., CT or FD). Intrinsic underestimation of tumour size on CT and comparatively good agreement of FD measurements is apparent as is the size-dependent increase in volume underestimation on CT. In **c** and **f**, an intra-individual comparison of the methods is shown to visualise the consistent size underestimation for both volume and maximum diameter measurements

**Fig. 3:**
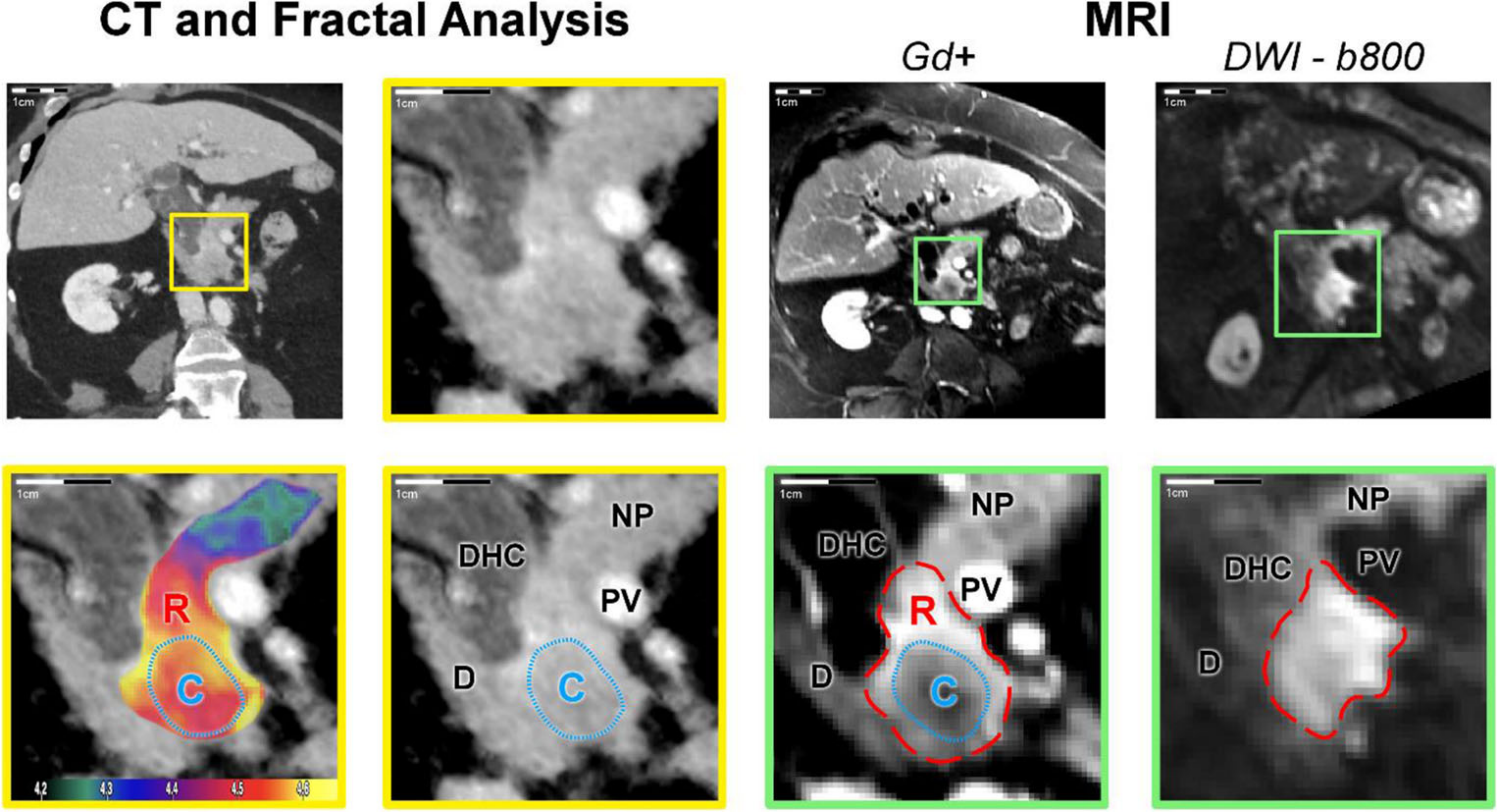
Representative example of pancreatic ductal adenocarcinoma in the uncinate process contiguous with the portal vein. The panel organisation is analogue to Figure 1. A paracoronal reformation was chosen to depict the blind truncation of the common bile duct (ductus hepato-choledochus, DHC) and involvement of the portal vein, which was not apparent on conventional CT. CT with fractal dimension (FD) map of perfusion (left and middle columns with yellow frames, FD colour-code given in the bottom inset) and MRI as reference. A contrast-enhanced, fat-saturated T1 VIBE image (acquired after administration of a gadolinium-based contrast agent) in venous phase (Gd+) is shown to illustrate tumour conspicuity alongside a diffusion-weighted image with *b*-value = 800 s/mm^2^ (DWI - b800), which was used for size estimation. The panels with yellow and green frames are magnifications of the tumour area in CT and MRI, respectively, and correspond to the marked areas in the images with original resolution. The hypoperfused tumour core (C, blue dotted line) is depicted in CT and MR (Gd+) images. On CT, no tumour extension to the portal vein was suspected. However, the tumour rim extends into the pancreatic head as seen on MRI and in the FD map (R, high FD mapping values or red dashed line), whereas CT only shows the tumour portion in the uncinate process. Tumour volume was 10 ml on CT, 19 ml on FD map, and 20 ml on MRI. Conventionally measured tumour diameter was 24 mm on axial slices, whereas Feret’s calculated maximum diameter along the longest axis in arbitrary orientation was 32 mm on CT, 47 mm on FD map, and 50 mm on MRI. C, tumour core; R, tumour rim; NP, non-tumourous pancreatic tissue; PV, portal vein; D, duodenum; DHC, ductus hepato-choledochus

**Fig. 4:**
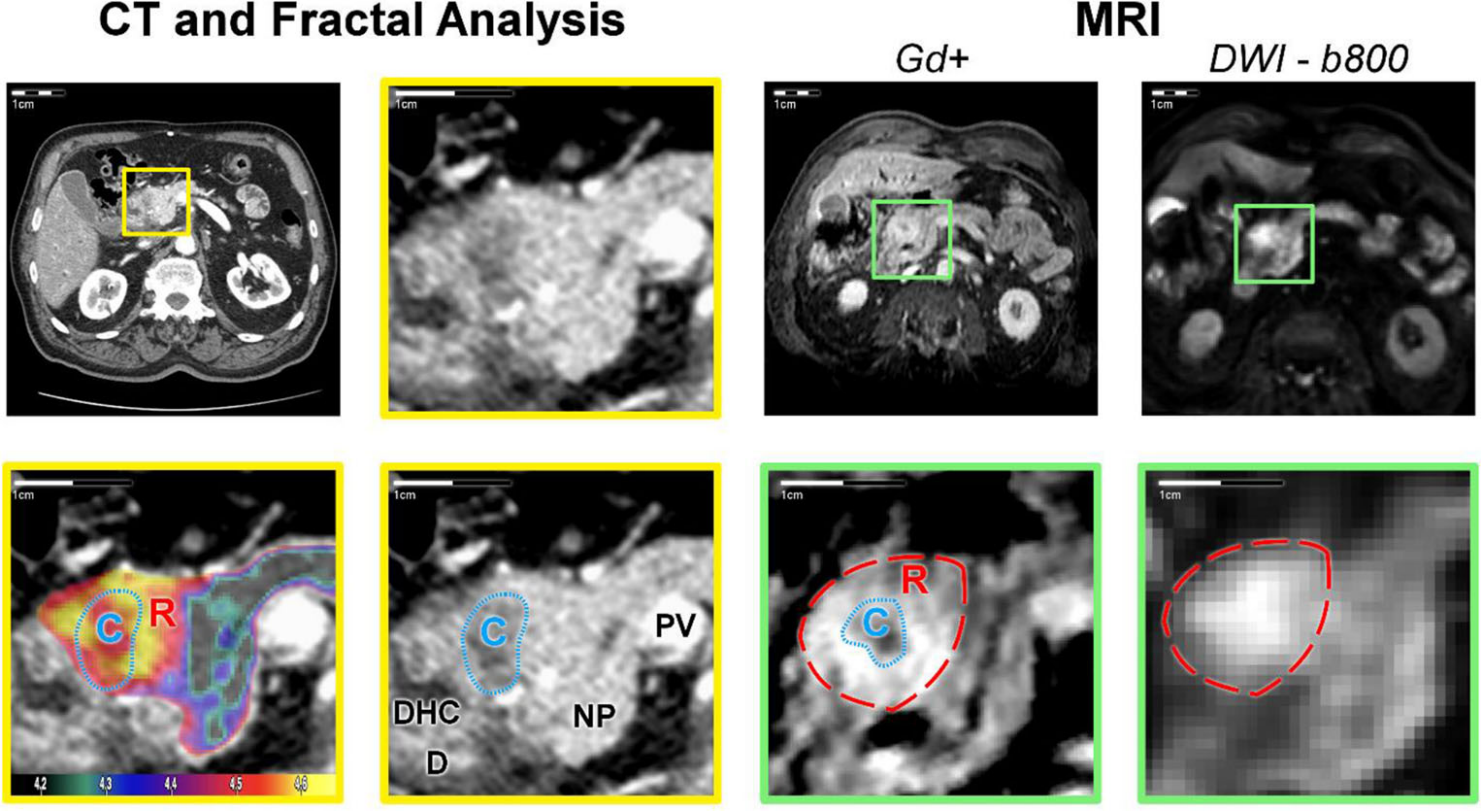
Representative example of pancreatic ductal adenocarcinoma in the pancreatic head. A para-axial reformation was chosen to simultaneously depict FD differences in the tumour core, rim, and adjacent non-tumourous pancreatic tissue. The panel organisation is analogue to Figure 1. On CT, the tumour core is the only visible portion of the tumour. In contrast, the tumour rim (R, high FD mapping values or red dashed line) is well depicted on MRI and FD maps and its perfusion pattern is more chaotic than in the tumour core and adjacent non-tumourous pancreatic tissue as indicated by the FD. Tumour volume was 3 ml on CT, 8 ml on FD map, and 9 ml on MRI. Tumour diameter was 18 mm on axial slices, whereas Feret’s calculated maximum diameter along the longest axis in arbitrary orientation was 20 mm on CT, 33 mm on FD map, and 29 mm on MRI. C, tumour core; R, tumour rim; NP, non-tumourous pancreatic tissue; PV, portal vein; D, duodenum; DHC, ductus hepato-choledochus

For diameter measurements, FD-derived maximum tumour diameters agreed well with MRI reference (Δdiameter = 1.5 mm, *p* = 0.63), while maximum tumour diameters were consistently underestimated on CT (Δdiameter = 10.6 mm, *p* < 0.001). None of the diameter measurement methods showed an evident dependence on the actual tumour diameter (FD: *p* = 0.84, CT: *p* = 0.95). In a subgroup of 20 PDA lesions, inter-reader agreement was excellent both for diameter measurement (mpMRI—median diameter discrepancy: 3.9%, interquartile range [IQR]: 10.8%, Spearman’s *ρ* = 0.99; FD—median diameter discrepancy: 0.1%, IQR: 10.8%, *ρ* = 0.96; CT—median diameter discrepancy: 4.8%, IQR: 12.4%, *ρ* = 0.92) and for volume measurement (mpMRI—median volume discrepancy: 1.1%, IQR: 9.1%, *ρ* = 0.99; FD—median volume discrepancy: 4.5%, IQR: 6.5%, *ρ* = 0.99; CT—median volume discrepancy: 4.4%, IQR: 27.3%, *ρ* = 0.98). Detailed statistics on correlation and bias of the methods, including Bland-Altman plots, can be found in Figure 2 and Table 3. Two representative cases are presented in Figures 3 and 4.

**Table 3:** Comparison of volume and maximum diameter measurements by method with multi-parametric MRI as reference standard in the validation cohort

Method	Bland-Altman statistics			Linear correlation with reference	
	Mean difference (lower and upper limit)	Slope of regression line (CI)	Intercept of regression line (CI)	Slope (CI)	*R*^2^
Volume measurements (ml)					
FD	0.61 (−3.09 to 4.31)	0.05 (0.01 to 0.09)	−0.44 (−1.50 to 0.63)	0.95 (0.91 to 0.98)	0.99
CT	10.19 (−3.53 to 23.90)	0.46 (0.33 to 0.58)	2.66 (0.15 to 5.16)	0.59 (0.51 to 0.67)	0.87
Diameter measurements (mm)					
FD	−1.54 (−8.36 to 5.29)	0.03 (−0.06 to 0.12)	−3.16 (−7.58 to 1.25)	0.94 (0.85 to 1.02)	0.93
CT	10.59 (−1.22 to 22.41)	0.01 (−0.15 to 0.17)	10.15 (3.33 to 16.98)	0.89 (0.75 to 1.04)	0.81

Absolute volume or diameter measurements are given in ml or mm, where applicable. *FD*, fractal dimension map; *CI*, 95% confidence interval; *R*^*2*^, coefficient of determination

### Perfusion chaos in the tumour rim

**Fig. 5:**
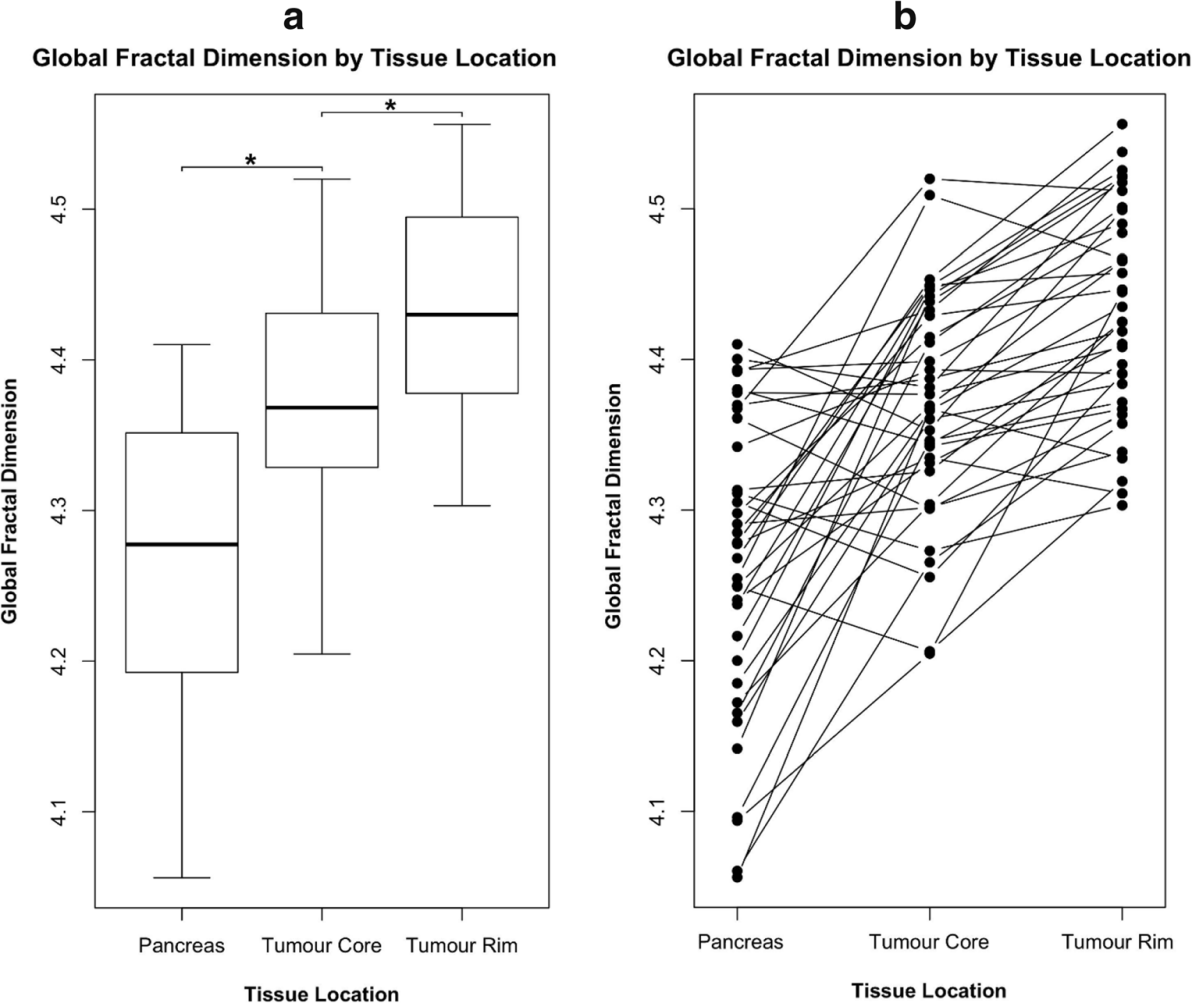
Global fractal dimension (FD) by anatomical location aggregated in a boxplot (**a**) and in intra-individual comparison (**b**). The global FD was lowest in adjacent non-tumourous pancreatic tissue, intermediate in the core of pancreatic ductal adenocarcinomas, and highest in the tumour rim as visualised on local FD maps. *—significance level *p* ≤ 0.003

To test the pathophysiological hypothesis of high perfusion chaos in the tumour rim, we performed an additional global fractal analysis of the tumour rim, the tumour core, and adjacent pancreatic tissue (Figure 5). In contrast to the local FD maps as reported above, the global FD yields a scalar value, which quantitatively summarises the overall chaos in a ROI. The global FD was significantly (*p* ≤ 0.003) higher in the tumour rim (global FD_rim_ = 4.43, [quartiles: 4.38–4.49]) than in the tumour core (global FD_core_ = 4.37 [quartiles: 4.33–4.43]), and both were higher than the FD in remote pancreas tissue (global FD_pancreas_ = 4.28 [quartiles: 4.19–4.35]); i.e., perfusion was highly chaotic in the tumour rim, less chaotic in the tumour core, and least chaotic in remote pancreas tissue (Figure 5). Interestingly, CT was hardly, if ever, capable of depicting the tumour rim visually, which is evident from measured tumour volumes and diameters.

## Discussion

In patients with PDA, we found evidence that CT consistently underestimates tumour size, while mpMRI allows realistic tumour size measurements in comparison to gross pathology measurements. Fractal analysis of routine contrast-enhanced CT imaging data consisting of unenhanced, parenchymal, and venous enhancement phases allowed to improve accuracy of tumour size estimation on CT in comparison to measurements on gross pathology and mpMRI. Our study shows that perfusion is more chaotic in tumours compared to remote pancreas tissue and has a tumour rim-to-core gradient, which can be exploited for more realistic size estimations. The vital tumour rim is incompletely visualised on plain CT images and thus does not contribute to conventional tumour size estimates. Conversely, fractal analysis improves the depiction of the tumour rim based on the amount of chaos in the perfusion pattern and thus allows its reliable identification.

Our results document a systematic underestimation of tumour diameter on CT and a good agreement of mpMRI measurements in comparison to gross pathology examination. Usually, tumour diameters are measured in one of the three perpendicular standard planes. In our study, however, we measured the largest diameter three-dimensionally, which we derived from volumetric tumour segmentation by calculating Feret’s diameter. This approach ensures a more realistic representation of the maximum diameter because it is independent of the actual orientation of the longest axis in space. CT measurement of PDA size is prone to underestimation not only because of poor visualisation of the tumour rim but also because of possibly tilted tumour orientation. Therefore, the tumour is not properly represented in the three perpendicular standard planes and might be measured in an inadequate plane.

Since perfusion and vascularity are a hallmark of cancer, tumour blood supply plays a crucial role in tumour development and progression including its ability to perforate basal membranes and to extend beyond organ borders as well as its metastatic potential. We believe that chaos of perfusion—as quantified by the FD—reflects the vascular aspect of tumour pathophysiology and might yield insights into its biological state. Specifically, tumour neoangiogenesis patterns in PDA have been previously characterised histopathologically, and higher vascular density in the tumour rim relative to the core has been observed [15–17]. This phenomenon has been attributed to activation of pancreatic stellate cells in the tumour rim, which are profibrogenic stromal cells and play a role in upregulating angiogenesis in the rim [20]. These histopathological findings explain the higher perfusion rates in the rim of PDAs compared with their cores as observed in previous radiological studies [18, 19]. Our results additionally indicate an increase in perfusion chaos, which can be exploited for visualisation of tumour extent, thus improving size measurement on CT. Moreover, we observed that size discrepancies in tumour volumetry increased with absolute tumour volume, which tended to be associated with the presence of a larger rim not apparent in conventional CT.

Further research might investigate whether the FD, as a quantitative imaging biomarker, is suitable to guide clinical management with respect to therapy planning and patient outcome. Similar to other tumour applications (e.g., colorectal cancer, bronchial adenocarcinoma, glioma) [12], fractal analysis might have prognostic implications as it provides information on the vital tumour rim. Therefore, it should be investigated whether fractal analysis could also predict other important clinical characteristics such as metastatic potential or the likelihood of a response to neoadjuvant chemotherapy. The differentiation of viable tumour from dense fibrosis, which can occur in the periphery of PDA [31], constitutes another interesting aspect that might be addressed by further research. The perfusion pattern of dense fibrosis, which is probably associated with hypoperfusion and delayed enhancement, might be different from that of the viable tumour rim.

This study has limitations. We performed a single-centre study, and our results might therefore not be fully representative of clinical practice at other hospitals. However, we applied fractal analysis to a commonly used CT imaging protocol including unenhanced, pancreatic parenchymal, and portal-venous phases; however, some centres prefer to use CT protocols without an unenhanced scan. We did not investigate dual-energy CT (DECT), which has the potential to improve contrast-to-noise ratio and tumour conspicuity in low monochromatic energy images and iodine density images and allows reconstruction of virtual unenhanced images [32]. However, DECT is still not recommended in the guidelines for staging PDA and is not widely available. Some studies have reported size underestimation on MRI when compared to tumour size measured on resected specimens [33, 34]. Those studies used MRI sequences with the best conspicuity (namely T1-weighted post-contrast sequences), which have the same physiological drawback as contrast-enhanced CT scans in terms of size determination. Instead, we used T2-weighted and DWI sequences for size measurement using the approach described in [9]. Our retrospective study design did not allow a histological correlation with fractal properties of microvascular architecture. However, fractal analysis of microcirculation would require three-dimensional microvessel segmentation and volumetric inter-slice registration of histological slices, which is very challenging and—to our knowledge—has not yet been established for PDA. Despite the small number of patients (*n* = 46), our study cohort is still representative of a variety of sizes and common clinical conditions found in PDA. However, our study does not represent all clinical circumstances, including the condition after neoadjuvant chemotherapy or very small tumours (< 1 cm in diameter). Moreover, our study did not include extremely obese patients, in whom a more extensive denoising scheme might be required. Rigorous denoising might affect image texture and thus degrade analysis of perfusion chaos. Since only a mild denoising scheme with noise-level adaptation was required in our study, we were able to preserve biology-induced texture. This might not be possible in extremely obese patients. We performed conventional diameter measurement according to RECIST recommendations to characterise the tumours. However, all further analyses were carried out using Feret’s diameter obtained from 3D tumour volumetry. Feret’s diameter is less prone to inter-observer variability and represents a more realistic estimate of tumour diameter because it is independent of the spatial orientation of both the tumour and its longest axis [27].

In summary, our study establishes fractal dimension as an imaging biomarker that improves size measurement of PDA in CT imaging based on visualising perfusion chaos. Our study demonstrates consistent underestimation of PDA lesion size, in terms of both diameter and volume, whereas FD maps calculated from CT correlated well with mpMRI and gross pathology. Chaos of perfusion might be a relevant pathophysiological aspect that potentially explains incomplete tumour depiction on CT imaging.

## Abbreviations

FD: Fractal dimension
mpMRI: Multi-parametric magnetic resonance imaging
PDA: Pancreatic ductal adenocarcinoma

## Declarations

### Guarantor

The scientific guarantor of this publication is Prof. Marc Zins.

### Statistics and biometry

No complex statistical methods were necessary for this paper.

### Methodology

• retrospective

• experimental

• performed at one institution
